# Functional Outcomes and a Review of Management Options for Revision Shoulder Arthroplasty

**DOI:** 10.5704/MOJ.2407.003

**Published:** 2024-07

**Authors:** AK Rai, K Kumar

**Affiliations:** Department of Orthopaedics, Woodend Hospital, Aberdeen, United Kingdom

**Keywords:** total shoulder replacements, shoulder revision surgery, outcomes, management

## Abstract

**Introduction:**

Increase in the number of primary shoulder arthroplasty has led to an increase in the number of revisions which presents many complex challenges and often has inferior outcomes.

**Materials and Methods:**

Data was collected retrospectively, and patients were classified using Dines classification. Comprehensive case reviews were done to identify preoperative and intra-operative challenges. The primary outcome measure was Oxford shoulder score (OSS). The secondary measures were range of motion (ROM) and patient satisfaction (very satisfied, satisfied, not satisfied or worse).

**Results:**

A total of 32 patients were identified with a mean age of 67.64 years and the most common cause of revision was a combination of bone and soft tissue failure (39.3%). All patients (n=8) with hemiarthroplasty had rotator cuff deficiency while patients with resurfacing had both rotator cuff failure and bony erosion. Four patients needed a proximal humeral osteotomy and six patients needed allograft reconstruction of the glenoid for bone loss. Twenty-one shoulders were revised to reverse total shoulder arthroplasty (TSA), 2 to anatomical TSA and 5 were left with cement spacer in situ. Mean duration of follow-up was 41.6 months. Mean OSS at the last follow-up was 26.88 with statistically significant improvement in ROM. There was no statistical difference in clinical outcomes (p>0.05) based on the type of primary prosthesis or cause of revision. A total of 70% patients were pain free. Patients with infection had inferior outcomes with a mean OSS of 17.

**Conclusion:**

Management of patients with failed shoulder arthroplasty is often challenging but has good clinical outcome except in infections.

## Introduction

Revision shoulder arthroplasty is well known to have inferior outcomes when compared to primary shoulder arthroplasty^1^. The revision surgery is more commonly done after a shoulder hemiarthroplasty than a total shoulder arthroplasty^2^. In the past there were high failure rates in metal backed component of total shoulder arthroplasty as well^3^. However, the new generation of glenoid implants have proven to have longer survivability and as a result outcomes following total shoulder replacement have improved remarkably as compared to hemiarthroplasty^4^. As a result, there has been a considerable increase in primary shoulder arthroplasties and consequently, in the number of revisions in the last decade as well^5^. Revision shoulder arthroplasty presents several unique and complex challenges that need to be addressed to achieve a satisfactory functional outcome^6-8^. Most of the literature available have evaluated the outcomes of revision arthroplasty for selected procedures like revision for a loose glenoid, conversion of a resurfacing shoulder arthroplasty or hemi-arthroplasty to a total shoulder arthroplasty. As a result, it becomes difficult to plan revision strategies when faced with multiple problems.

The objective of this study was to review the challenges, management options and outcomes following revision arthroplasty in a variety of patients with multiple surgical problems.

## Materials and Methods

This is a retrospective study of all revision shoulder arthroplasty procedures performed at our centre from 2010 to 2017. Approval was obtained from Local Ethics Committee. A detailed case notes and radiologic review was performed by analysing clinic letters, operation notes and radiographs. All the primary procedures were done at our centre. The indication of primary arthroplasty, type of prosthesis and causes of failure were identified. We classified the causes of failure as soft tissue, bone related, infection or a combination of these factors as defined by Dines *et al*^1^. Pre-operative radiographs were evaluated to assess degree of glenoid erosion as per Sperling *et al*^9^. Grade 1 is no erosion, Grade 2 is erosion limited to subchondral bone, Grade 3 is moderate erosion with medialisation, and Grade 4 is medialisation beyond the coracoid base. In case of TSA, the radiographs were studied for osteolysis around components. All the surgeries were done at one centre and done by one surgeon. Operative records were reviewed to identify any specific technical difficulties and if any additional procedures were done.

All patients were evaluated for infection with routine blood tests – full blood count and inflammatory markers. When these were within normal limits with radiological evidence of other causes of failure, patients were planned for single stage surgery. Samples were sent intra-operatively from all aseptic cases to rule out underlying infection. Deltopectoral approach was used in all the cases. Surgical exposure was challenging due to the presence of scar tissue from previous surgery. Axillary nerve was routinely identified and protected throughout the procedure. Glenoid erosion was assessed from pre-operative radiographs. In case of glenoid erosion of Grade 3 or more allograft was requested for glenoid bone grafting. Proximal humeral osteotomy was done if stem extraction was difficult. In cemented stems, it also facilitated extraction of residual cement. This was done manually using chisels and curettes. The osteotomies were secured with cables. In case of infection the strategy was to perform debridement with retention of implant if the infection was acute. In case of chronic infection, two stage revisions were planned with first stage involving removal of implant and replacement with an antibiotic cement spacer. At least five samples were sent for microbiology from different areas to help with targeted antibiotic therapy in postoperative period. A multidisciplinary team was involved in their management which included infectious disease specialist. They received intravenous antibiotics for at least eight weeks after which they were re assessed for second stage.

Post-operative radiographs of the most recent follow-up were reviewed for any radiolucency’s or scapular notching in cases of reverse shoulder arthroplasty. The primary outcome measure was Oxford shoulder score (OSS). The secondary measures were range of motion (ROM) and patient satisfaction (satisfied, equivocal or dissatisfied). Statistical analysis was done using Microsoft Excel.

## Results

A total of 32 patients underwent revision shoulder arthroplasty during the period 2010-2017. One patient had bilateral revision procedure and hence the total number of cases included in the study were 33.

The mean age of the patients was 67 years (Range 42-86). The most common symptoms were pain and stiffness (n=17, 51.5%). One patient did not have any symptoms but had some clicking on examination during routine follow-up. The radiographs revealed that the glenoid component was grossly loose (Table I).

**Table I:** Demographic and clinical characteristics of patients.

	Overall	Resurfacing	Hemiarthroplasty	TSA	Reverse TSA
Mean Age (Years)	67.1 (SD-12.2)	61.7 (SD-14)	73.4 (SD-7.7)	61.7 (SD-12)	72 (SD-10.5)
Sex					
Male (n)	13	4	4	2	3
Female (n)	20	7	6	4	3
Median time between primary and revision (months)	28 (1-240)	36 (13-72)	43 (10-120)	23 (1-240)	12.5(1-48)
Symptoms of patients (n)					
Pain and stiffness	25	11	9	3	2
Stiffness	1			1	
Pain and instability	1		1		
Acute pain	3				3
Wound problems	2			1	1
No symptoms	1			1	

Abbreviations – TSA: total shoulder arthroplasty, SD: standard deviation, n: number of patients

Eleven patients had resurfacing, 10 had hemiarthroplasty and 12 had total shoulder arthroplasty (TSA). The median duration between primary and revision procedure was 28 months (Range 1 – 240). The survival of TSA was 37.8 months, that of resurfacing was 37.6 months while for stemmed hemiarthroplasty it was 56.1 months.

The most common indication of primary arthroplasty was osteoarthritis (n=12) (Table IIa). The most common cause of revision (excluding infection) was a combination of bone and soft tissue failure (39.3%, n=13), while soft tissue failure alone was contributed in 21.2% (n=7) cases (Table IIb). All patients (n=9) with hemiarthroplasty had a component of rotator cuff failure evident by superior migration with only half of them (n=4) associated with bony erosions. On the contrary, rotator cuff failure and bony erosion were present in most patients who had undergone resurfacing (n=7). Most of the reverse TSAs had a bone related failure (2 with glenoid loosening and 2 with fractures). In two patients with resurfacing with chronic pain, an obvious cause could not be identified. It was presumed that their symptoms were related to glenoid wear and hence both were revised to anatomical TSA.

**Table II:** (a) Aetiology of primary arthroplasty. (b) Causes of failure. (a)

Primary Cause of arthroplasty	Overall (n)	Resurfacing (n)	Hemiarthroplasty (n)	TSA (n)	Reverse TSA (n)
Osteoarthritis	12	6	3	1	2
Inflammatory arthritis	7	4		3	
Fracture	7		7		
Avascular Necrosis					
Primary	1				1
Secondary	2	1		1	
Massive cuff tear	1				1
Rotator cuff arthropathy	2				2
Glenoid cyst	1			1	
Total	33	11	10	6	6

**Table:** (b)

	Resurfacing (n)	Hemiarthroplasty (n)	TSA (n)	Reverse TSA (n)
Soft tissue				
Rotator Cuff failure	9	8	2	0
Heterotopic Ossification	0	0	1	0
Bone failure				
Glenoid erosion > Grade 3	5	2	0	0
Glenoid erosion < Grade 2	3	2	0	0
Glenoid loosening	0	0	2	2
Periprosthetic fracture	0	0	0	2
Infection	0	1	3	2

Abbreviations – TSA: total shoulder arthroplasty, n: number of patients

**Fig. 1:**
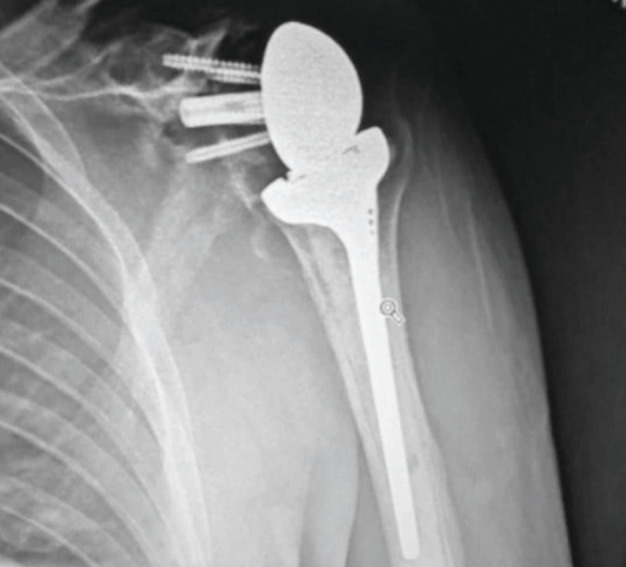
Bone graft for glenoid bone loss.

Patients who had a component of rotator cuff insufficiency due to soft tissue failure underwent reverse TSA. Overall, 21 shoulders were revised to reverse TSA and three to anatomical TSA. Two patients underwent only glenoid component revision. Two patients with reverse TSA had revision of the humeral stem to longer stems following a periprosthetic fracture. Post-operatively none of the samples in aseptic group showed growth of any organisms. All humeral head resurfacings that needed revision were uncemented. Four hemiarthroplasties were cemented while six were uncemented stems. Proximal humeral osteotomy was needed for one uncemented stem and one cemented stem. In case of anatomical total shoulder replacement, five were short stem anatomical TSA while one was with a long humeral stem. The latter needed proximal humeral osteotomy for stem extraction. In case of reverse TSA, four humeral stems were uncemented with one needing proximal humeral osteotomy. All proximal humeral osteotomies healed without any complications. On the glenoid side, two glenoid components had significant osteolysis pre-operatively and needed allograft augmentation. Four other patients needed allograft reconstruction of the glenoid for bone loss due to erosion. Allograft used was considered when the hold of the metaglene and glenoid screws was poor. A long pegged glenoid was used and the graft was stabilised using the screws for fixation of the metaglene (Fig. 1).

Pre-operatively, infection was diagnosed in 18% (n=6) of the patients with acute presentation (<3 weeks) in one patient while others had late presentation (>12 weeks) (Table III). Six patients were revised due to infection of which five were patients with TSA and one with a hemiarthroplasty. Interestingly, four out of six patients had history to previous surgery in the same shoulder. One patient had infection in the post-operative period with subscapularis failure. Multiple washouts were needed to control the infection and then, subscapularis reconstruction with a dermal allograft patch was attempted. However, the infection recurred, and prosthesis had to be replaced with antibiotic spacer. Others were planned for two stage revision surgery. Although a second stage was planned for all of them, only one patient underwent a 2-stage revision with reverse TSA. Others did not prefer to have any further surgery as their pain was controlled although with limited function. They remain in regular follow-up.

**Table III:** Revision in patients with infection.

S. No	Age	Primary diagnosis	Type of primary implant	Previous surgeries to Shoulder	Type of infection	Clinical details	Management
1	71/M	Osteoarthritis	Hemiarthroplasty	Yes	Chronic	Pain	Spacer for 8 weeks with IV antibiotics and rTSA in 2nd stage
2	77/M	Rotator cuff arthropathy	Reverse TSA	Yes	Chronic	Pain + Loosening of glenoid	Prosthesis replaced with cement spacer
3	49/F	AVN following malunited fracture	Anatomical TSA	Yes	acute	Wound dehiscence + instability	Wound washouts + IV antibiotics; when controlled subscapularis repair with arthroflex patch; replaced with cement spacer
4	76/F	Massive cuff tear	Reverse TSA	No	Chronic	Pain and abscess	Washout and prosthesis replaced with cement spacer + IV antibiotics
5	83/F	Osteoarthritis	Anatomical TSA	No	Chronic	Pain and stiffness	Washout and prosthesis replaced with cement spacer + IV antibiotics
6	53/F	Glenoid cyst	Anatomical TSA	Yes	Chronic	Pain and stiffness	Washout and prosthesis replaced with cement spacer + IV antibiotics.

Mean duration of follow-up was 41.6 months (2 - 104 months). One patient passed away during follow-up. Forward flexion (FF) improved from a mean of 43.20^0^ to 86.70^0^ and abduction improved from a mean of 41.60^0^ to 87.30^0^ which was statistically significant (p<0.005) using paired t-test. This was the case for all three groups of primary prosthesis (Table IV). Mean OSS at the last follow-up was 26.88. We also found that there was no statistical difference in clinical outcomes based on the type of primary prosthesis when all three types of prosthesis were compared using one way ANOVA test for ROM (p=0.25 and p=0.45) and OSS (p=0.28). Also, there was no statistical difference in ROM (p=0.4 and p=0.29) and OSS (p=0.69) based on the cause of revision – whether bone related, soft tissue or both with infection group excluded. A total of 23 (70%) patients reported that they were pain free and satisfied with the function. Four patients felt that their pain was worse than before and were dissatisfied with revision procedure. Three of these were from the resurfacing group while one was from infected group. No cause could be identified, and patients didn’t want any further surgery. We also noted that those who continued to have pain post-operatively had a mean age of 70.4 years which was higher than the mean age of the cohort. This also included two patients with age less than 60 indicating that young or very old patients may not achieve the best clinical outcome. In case of revision following infection however, there was a mean improvement in FF and abduction from 31.70° to 53.30° and 38.30° to 55° which were both not significant statistically. They also had a mean OSS of 17 which was lower than all other causes of revision which was statistically significant (p=0.009) calculated using unpaired t-test.

**Table IV:** Functional outcome.

Patient group	Mean Forward Flexion (degrees)			Mean Abduction (degrees)			OSS	Number of patients
	Pre-op	Post-op	p-value	Pre-op	Post-op	p-value		Pain free
Resurfacing	40.91 (SD-31.1)	103.64 (SD-40.1)	0.0006	40.45 (SD-26.5)	99.09 (SD-34.8)	0.0003	27.36 (SD-12.9)	6
Hemiarthroplasty	32 (SD-18.1)	79 (SD-36.7)	0.0030	32 (SD-17.5)	83 (SD-32.3)	0.0006	31.22 (SD-9.6)	8
TSA	38.46 (SD-39.6)	77.5 (SD-42.6)	0.0367	38.46 (SD-33.6)	80 (SD-43)	0.0202	23.17 (SD-10.5)	9
Overall (excliudng infection)	37.58 (SD-33.7)	86.67 (SD-38.5)	<0.001	37.42 (SD-26.1)	87.27 (SD-34.1)	<0.001	26.88 (SD-12.4)	23
Infection	31.67 (SD-30.6)	53.33 (SD-34.4)	0.28	38.33(SD-29.9)	55.00(SD 31.4)	0.37	17 (SD-6.7)	4

We had an overall complication rate of 18% (n=6). Two patients had dislocations and both patients underwent another procedure – one for revision of glenoid component while other had humeral stem revised. Two patients had neuropraxia, one of axillary nerve and other of posterior interosseus nerve with both resolving during follow-up. One patient had hematoma which needed evacuation postoperatively and another patient had fracture of the humerus intra-operatively which was fixed with a DCP intra-operatively. At the end of the follow-up, in case of reverse TSA there were s/o radiolucency in 5 (20%) glenoid prosthesis and 9 (36%) humeral stems. Fifteen (60%) patients had evidence of scapular notching, with 10 having only Grade 1. One patient with anatomical TSA did not have any radiolucencies. There was severe stress shielding of the humeral stem noted in one patient.

## Discussion

Failure of arthroplasty is defined as patient dissatisfaction following the procedure, regardless of the severity of symptoms or physical findings^9-10^. Hasan *et al*^10^ and Franta *et al*^11^ found pain and stiffness to be the most common symptom which was similar to this study (n=17, 51.5%). Pernes *et al*^12^ have in the past advocated that pain should primarily guide the decision towards revision surgery.

Failure following shoulder arthroplasty can be attributed to more than one cause^1,9,10^. In this study, soft tissue failure either in isolation or in combination with other factors was the most common mode of failure in stemmed hemiarthroplasty and resurfacing. Glenoid erosion was more commonly seen in the latter. This contrasts with Gaeremynck *et al*^13^ and Jaiswal *et al*^14^ who have suggested that bone erosion is the primary cause of failure in revision surgeries. The data from UK national joint registry, however, suggests that rotator cuff insufficiency is the main cause of revision arthroplasty^5^. This is also in agreement with Kelly *et al*^15^ who concluded that rotator cuff insufficiency was more common in their series of 38 patients. Knowles *et al*^16^ in their systematic review noted that rotator cuff insufficiency is the most common cause of revisions in shoulder arthroplasty. If there is no superior migration on the radiographs, rotator cuff should be assessed with further imaging and intra-operatively. If it is found to be competent then anatomical TSA may be used, as was the case in one patient in our study group.

Hemiarthroplasties are usually done in cases of fractures, and it is often difficult to reconstruct the tuberosity at the time of primary prosthesis. They are the most common type of prosthesis which are revised (up to 47%) across North America and Europe^16^. This could result in malunion, non-union or resorption of tuberosity and in turn leads to rotator cuff insufficiency^17^. This warrants some unique considerations during revisions. Hackett *et al*^17^ have suggested that abnormal morphology of tuberosities leads to lack of a structural support and landmarks for the humeral component positioning which should be considered in preoperative planning. As it is difficult to reliably overcome this hurdle, the choice of revision implant is reverse TSA which also helps in overcoming rotator cuff insufficiency. This is most likely the reason for reverse TSA being the most common arthroplasty type used in revision procedures^16^.

Bone loss is another challenge which needs to be addressed in revision surgery. Several authors^6,7,12^ in the past have stressed that the quality and quantity of bone stock is paramount in revision surgery. On the glenoid side, bone loss could be due to glenoid erosion in case of resurfacing/hemiarthroplasty or because of loosening of the glenoid component in case of TSA. Scapular notching could also contribute to bone loss in reverse geometry TSA. Preoperative metal artefact reduction CT scan should be done in cases when a severe bone loss is suspected^7^. Mild to moderate bone loss (Grade 2-3) can be dealt with concentric reaming, excision of a high anterior or a posterior side^6^ or by altering the orientation of the glenoid component to take advantage of good bone stock^7^. Most of the implants are available with longer peg options to take advantage of native bone deeper within the vault. In our experience, bone graft is useful in revision of TSA or if glenoid erosion is at least Grade 4 which is in agreement with other authors^6,18^. This study has shown that some cases of Grade 3 erosion can be managed without bone graft. We recommend that if the glenoid peg has a good hold a graft may not be needed. Dines *et al*^6^ have suggested that bone grafting can be done as a staged procedure to allow for incorporation of bone graft. However, single stage allograft reconstruction was done in all cases in this study without any problems in the follow-up. Sometimes the glenoid cannot be reconstructed and a hemiarthroplasty can be performed with reduced functional expectations^18^.

**Fig. 2:**
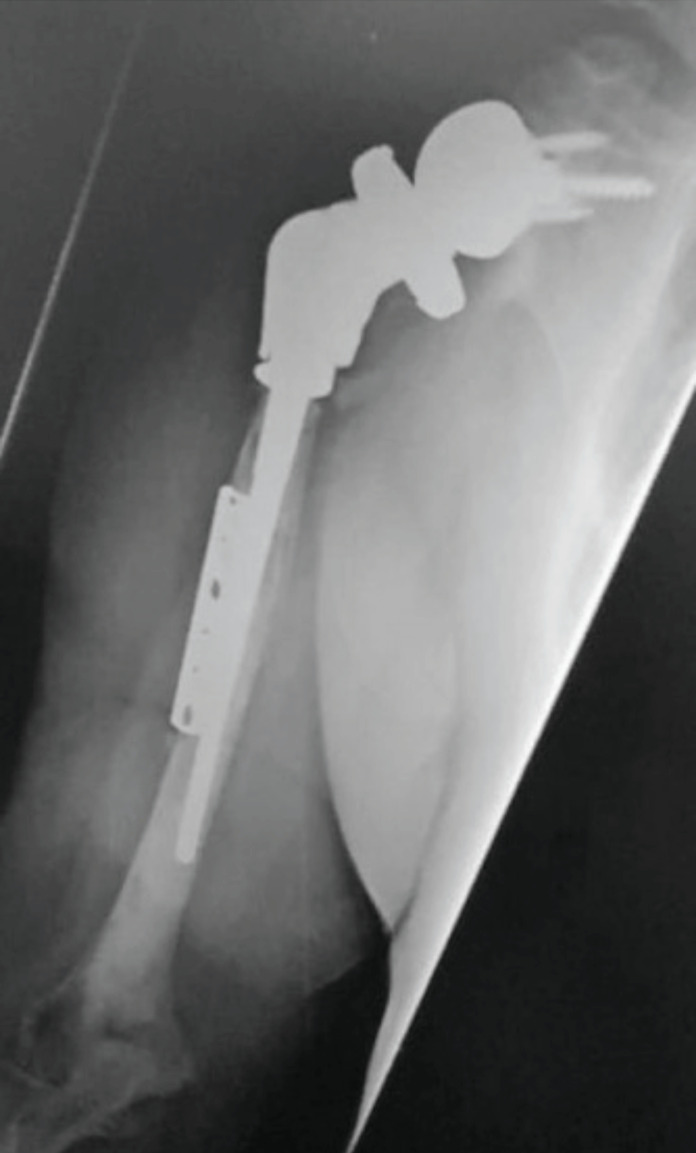
Long humeral stem with metaphyseal support for humeral bone loss.

On the humeral side, the bone loss can occur because of resorption of tuberosities, infection or osteolysis from polyethylene debris. Occasionally, it may be caused during the process of removing a well-fixed humeral prosthesis^7^. Aseptic humeral component loosening is a rare cause of arthroplasty failure^6,14^ and this has been reinforced in this study. Allograft could be used when bone loss involves the entire metaphysis^18^. Garver *et al*^7^ have suggested that humeral bone loss results in lack of structural support and altered deltoid kinematics advocating the use of allograft-reverse shoulder prosthesis composite (APC). APC can shield the implant from rotational stresses and provide soft-tissue attachment sites for subscapularis repair but has the downsides of allograft use, increased cost, and complexity of the procedure. A custom-made stem for metaphyseal support can also be used to address this problem which is less complex than APC (Fig. 2). Kelly *et al*^15^ have described using tricortical iliac crest bone graft for bone loss on both the humeral and glenoid side but long-term follow-up are not available to support their use. It can sometimes be difficult to remove a well-fixed humeral stem and a humeral osteotomy may be required^12,15^. Four patients in this study needed an osteotomy which was lower than in the series by Sheth *et al*^19^. The osteotomy was fixed using cables and healed without any complications.

Reimplanting the stem in the presence of bone loss poses a challenge due to the loss of anatomical landmarks^17^ leading to inferior instability if it is too low and superior impingement or instability if it is too high^6^. Judgement on appropriate height and tension is generally based on the experience of the surgeon while using the trial humeral stem. As with the standard reverse arthroplasty, the only guide is that the reduction should feel ‘tight’^20^. Uncemented stems may be used if the humeral side is unaffected^18^ as was the case in three patients in this study.

**Fig. 3:**
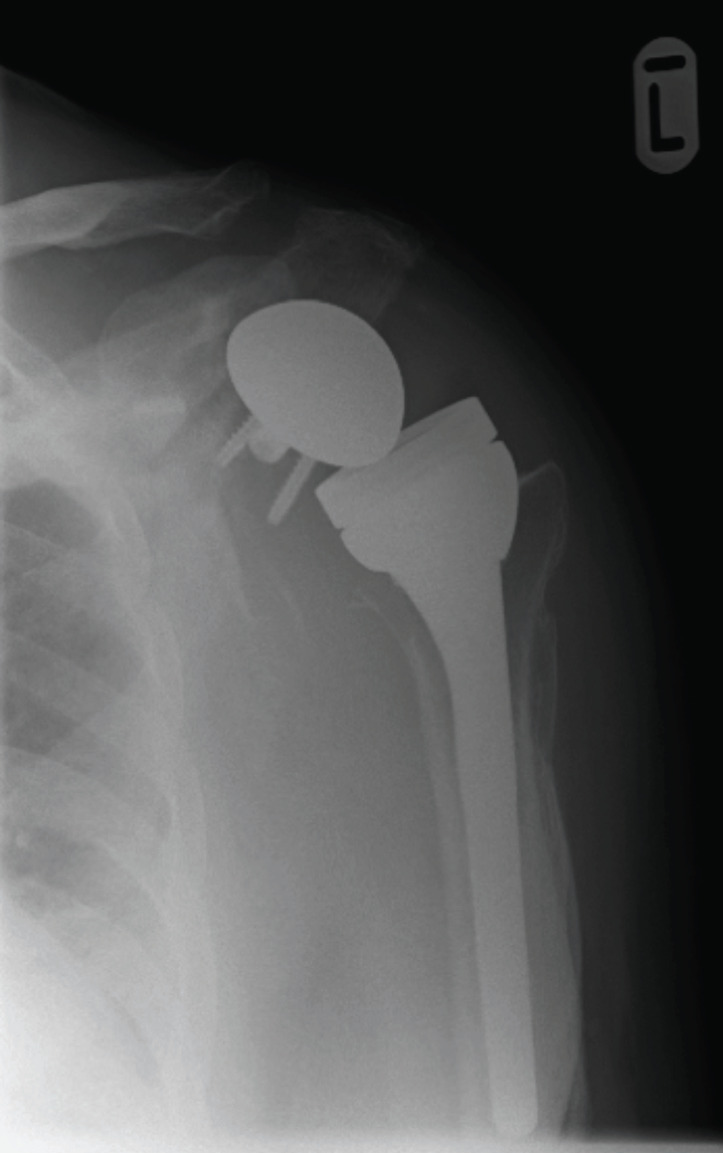
Loose glenoid component following infection.

The rate of infection in shoulder arthroplasty is usually low but when present can have poor outcomes^21,22^. Dines *et al*^1^ have divided all possible causes of revision into nine cohorts and found that the cohort with infection had the worst outcomes which is similar to findings of our study. The presentation is nonspecific with the most common symptom being pain^21,22^. The infection can be – acute (<3 weeks), subacute (3-12 weeks) and late (>12 weeks)^22^. Strickland *et al*^22^ have emphasised that it is wise to consider infection in the presence of a loose glenoid component which was the case with one patient in this study (Fig. 3). In our experience, management of infection should involve an infectious disease specialist. In acute infections washout with change of liner is an option^18^. In this study one patient with acute infection had serial washouts but patient continued to have persistent wound discharge. Consequently, the implant was removed and after thorough debridement an antibiotic loaded cement spacer was inserted.

In subacute or chronic infections some authors^6,7,22,23^ have suggested pre-operative aspiration. However, a negative aspiration does not specifically rule out infection^24^. There is insufficient literature regarding recommendations for one/two stage revisions or resection arthroplasty. A past history of shoulder surgery should alert the surgeon of risk of infection. In addition to prior surgery Nezwek *et al*^25^ have found that rheumatoid arthritis may also be associated with infection. The dilemma the surgeon faces in the presence of infection is that a spacer is necessary to control the infection, but two stage revisions have poorer outcomes^22^. In fact, the reinfection rate is very high despite two stage surgery^22^ making it difficult to justify the need for second stage surgery. Resection arthroplasty is also one of the options which has been suggested but it is not a preferred option in most cases as demonstrated in the review by four out of five patients in this study were satisfied with the spacers as they were pain free and did not want to go ahead with further procedures. They have remained pain free during follow-ups but of course with poor function. This is a bargain and avoids the need of a second surgery, thereby avoiding further complications. One patient who was not satisfied was referred to another centre for consideration of custom-made glenoid prosthesis due to the presence of severe glenoid bone loss from previous benign glenoid cysts.

Most authors agree that there is significant improvement in functional outcome following revision arthroplasty. However, the outcome is not comparable to primary arthroplasty^1,14,19,21,26^. In their study of 73 patients with a mean follow-up of 2 years Sheth *et al*^19^ concluded that there was an improvement in functional scores and ROM. A total of 22% of their patients, however, were dissatisfied which is similar to our study (30%). Kelly *et al*^15^ in their prospective study of 38 shoulders also had statistically significant improvement in forward flexion and abduction.

We also found that the outcome does not depend on the type of primary prosthesis. This is interesting because the challenges vary based on the type of implant. For instance, resurfacing hemiarthroplasties are done following arthritis and hence complications related to tuberosity are not encountered. Also, they are stemless implants and hence are not associated with bone loss as compared to stemmed hemiarthroplasties and TSA. Resurfacing and hemiarthroplasties are associated with glenoid erosion which can generally be managed without the need of bone graft if not severe. Total shoulder arthroplasties on the other hand are associated with more significant bone loss due to the presence of glenoid component. In our series 12 patients had glenoid erosion but only 3 needed a bone graft. However, three out of four patients with a loose glenoid component needed a bone graft. Most of the available literature is usually following a single type of primary prosthesis and studies comparing revisions following different types of primary implants are lacking.

The complication rate in our series was comparable to those by Sheth *et al*^19^ but it was less than average rate of complications in review by Saltzman *et al*^27^. Most authors agree that complications in revision arthroplasty is higher than in primary arthroplasty^27,28^. Dislocation can be because of subscapularis insufficiency, inadequate muscle tensioning or component mal-reduction^29^. In case of failure of conservative treatment or component mal-reduction, a revision surgery is warranted. One patient in this series had a component mal-reduction leading to dislocation while other had inadequate soft tissue tension. In case of recurrent dislocations, a salvage procedure like excision arthroplasty may be the only option^29^. Neuropraxia is usually a result of brachial plexus traction, prolonged retraction, and increased length of the procedure, can be managed conservatively^29^. Intra-operatively, fractures can occur during extraction or implantation of the prosthesis. One patient had an intra-operative fracture below the tip of the stem during extraction. The fracture was fixed with compression plate and arthroplasty was done as a second stage after healing of the fracture. Rahmi *et al*^29^ have suggested that often wiring is sufficient but sometimes a cemented stem may be necessary.

There are some limitations in this study-retrospective design, a small heterogenous sample size and non-availability of preoperative OSS. However, this is a single centre study of an uncommon procedure which is likely to increase in number in the future. The study does reflect the complexity and characteristics of patients undergoing revision arthroplasty which a shoulder surgeon is likely to encounter. The heterogeneity of cohort represents the variety of challenges the surgeon can encounter. It reviews the approach for management of these challenges and the surgical options available in recent literature.

## Conclusion

Management of patients with failed shoulder arthroplasty is often challenging with multiple causes of failure. It is necessary to carefully assess these factors as they can vary based on the primary implant. The options available are limited but if used appropriately can help achieve satisfactory outcome. However, the outcomes do not depend on the mode of failure or type of primary prosthesis but are significantly inferior in those undergoing revision due to infection.
